# In Vitro Evaluation of Pro- and Antioxidant Effects of Flavonoid Tricetin in Comparison to Myricetin

**DOI:** 10.3390/molecules25245850

**Published:** 2020-12-11

**Authors:** Vladimir Chobot, Franz Hadacek, Gert Bachmann, Wolfram Weckwerth, Lenka Kubicova

**Affiliations:** 1 Division of Molecular Systems Biology, Department of Functional and Evolutionary Ecology, Faculty of Life Sciences, University of Vienna, Althanstrasse 14, A-1090 Vienna, Austria; gert.bachmann@univie.ac.at (G.B.); wolfram.weckwerth@univie.ac.at (W.W.); lenka.kubicova@univie.ac.at (L.K.); 2 Department of Plant Biochemistry, Albrecht-von-Haller Institut, Georg-August-Universität Göttingen, Justus-von-Liebig-Weg 11, D-37077 Göttingen, Germany; franz.hadacek@biologie.uni-goettingen.de; 3 Vienna Metabolomics Center (VIME), University of Vienna, Althanstrasse 14, A-1090 Vienna, Austria

**Keywords:** aging, electroanalytical methods, Fenton reaction, free radicals, homeostasis, iron, neurodegeneration, polyphenols, reactive oxygen species, ROS

## Abstract

Flavonoids are rather common plant phenolic constituents that are known for potent antioxidant effects and can be beneficial for human health. Flavonoids with a pyrogallol moiety are highly efficient reducing agents with possible pro- and antioxidant effects, depending on the reaction milieu. Therefore, the redox properties of myricetin and tricetin were investigated by differential pulse voltammetry and deoxyribose degradation assay. Tricetin proved to be a good antioxidant but only showed negligible pro-oxidant activity in one of the deoxyribose degradation assay variants. Compared to tricetin, myricetin showed pro- and antioxidant effects. The more efficient reducing properties of myricetin are probably caused by the positive mesomeric effect of the enolic 3-hydroxy group on ring C. It is evident that the antioxidant properties of structurally similar flavonoids can be converted to apparent pro-oxidant effects by relatively small structural changes, such as hydroxylation. Since reactive oxygen species (ROS) often serve as secondary messengers in pathological and physiological processes in animal and plant cells, the pro- and antioxidant properties of flavonoids are an important part of controlling mechanisms of tissue signal cascades.

## 1. Introduction

In nearly all organisms, antioxidants represent an important tool to control cellular redox chemistry in attempts to maintain redox equilibrium [1]. This equilibrium (homeodynamics) includes primarily the buffering or slight modulation of reactive oxygen species (ROS) concentrations that participate in many physiological and pathological processes [1]. ROS levels are regulated by various enzymatic and non-enzymatic mechanisms. The latter involves also low-weight molecules with pronounced redox properties, such as polyphenols, ascorbic acid, tocopherols, and carotenoids [2,3]. While human beings can biosynthesize some low-weight redox-active molecules, such as kynurenines [4], the majority of the required antioxidants, ascorbic acid or polyphenols amongst others, needs to be taken up from food [4].

For many decades, polyphenols have been investigated extensively because of their beneficial effects on human health [5,6,7], especially flavonoids, a group of ubiquitously occurring plant polyphenols, well-known for their protective activities against degenerative diseases [6,8,9,10]. Assumedly, these effects depend probably both on ROS scavenging and coordination complex formation with iron [9,10]. Various flavonoid derivatives are clinically used for the treatment of angiopathies, such as diabetic microangiopathy, often initiating diabetic nephro-, retino-, and neuropathies [11,12,13]. Albeit flavonoid bioavailability from the human digestive system is limited, their positive effects on peptide ulcers and brain-gut axis have been discovered.

**Figure 1:**
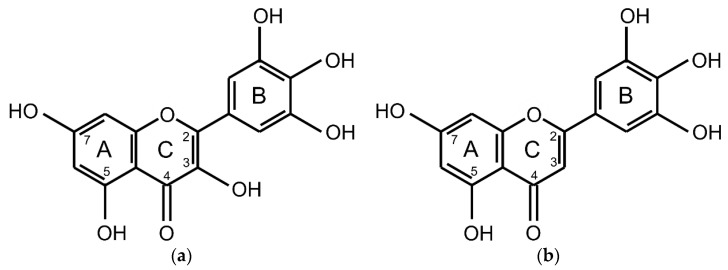
Chemical structures of (**a**) myricetin; (**b**) tricetin.

Within flavonoids, antioxidant activities differ in relation to the number and positions of hydroxy groups, especially those located on ring B; a catechol or pyrogallol arrangement of ring B enhances the flavonoid antioxidant activity [14]. Moreover, an enolic 3-hydroxy group located on ring C affects remarkably the observable ROS scavenging effects [15]. Accordingly, the presence of a pyrogallol moiety turns myricetin and tricetin (Figure 1) into renowned ROS scavengers.

Myricetin is well-known for its anticancer [16,17], hepato- [18,19], or neuroprotective activity [17,19], and preventive effects against cardiovascular diseases [17,19,20]. However, both anti- and pro-oxidant activities have been reported for this molecule [21]. Tricetin has been explored regarding anti-inflammatory [22,23], anticancer [24,25], antidiabetic [26], and possible neuroprotective [27] effects. Contrary to myricetin, the possible pro- or antioxidant effects of tricetin have not been investigated sufficiently yet.

Therefore, we have compared the antioxidant properties of tricetin with the structurally similar flavonoid myricetin, which possesses an additional enolic hydroxy group on position 3 of ring C. For this investigation, we have used the effective combination of differential pulse voltammetry (DPV, an electroanalytical method) with variants of the deoxyribose degradation assay (a chemical method). DPV has been successfully employed in the exploration of redox properties of various natural products, among them vine, vegetable, and fruit constituents [28].

The deoxyribose degradation assay utilizes 2-deoxyribose that is oxidized by hydroxyl radicals to thiobarbituric acid reactive species (TBARS). The hydroxyl radicals are generated by a Fenton-like reaction (Reaction 1).

H_2_O_2_ + Fe^II^→ Fe^III^ + ^––^OH + ^•^OH (Reaction 1)

After a reaction with thiobarbituric acid, the TBARS are quantified photometrically. The assay details and advantages of its variants are described elsewhere [29,30]. Compared to other assays, such as 2,2-diphenyl-1-picrylhydrazyl radical scavenging (DPPH assay) or ferric reducing antioxidant power (FRAP), the deoxyribose degradation assay informs both about possible antioxidant and pro-oxidant effects. These depend on the tested substance reactions with ROS, especially with hydrogen peroxide but also with molecular oxygen. Additionally, the deoxyribose degradation assay can discover a possible inhibition or promotion of iron redox cycling by the tested substances [29,30]. Since iron redox cycling is a key factor for Fenton-like reaction rates, the deoxyribose degradation assay explores an additional factor compared to most other applied assays that can affect the pro- and antioxidant activities of tested substances.

## 2. Results

### 2.1. Differential Pulse Voltammetry

**Figure 2:**
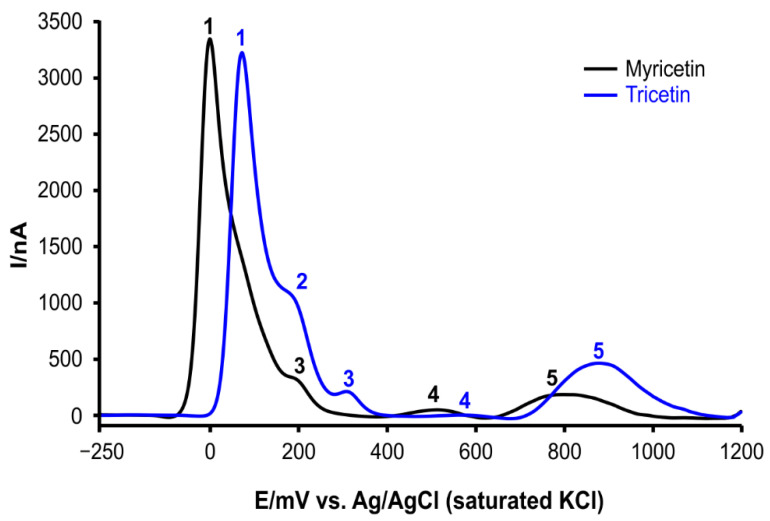
Differential pulse voltammograms of flavonoids myricetin and tricetin. 0.1 mM flavonoid solution in 0.1 M phosphate buffer (pH 7.4) with 10% (*v*/*v*) methanol. (Glassy carbon working electrode; platinum counter electrode; Ag/AgCl (saturated aqueous KCl solution) reference electrode; effective scan rate: 21 mV/s; modulation time: 0.05 s; modulation amplitude: 25 mV).

The voltammograms were recorded with a three-electrode system; the effective scan rate was 21 mV/s. Myricetin or tricetin was dissolved in a 0.1 M phosphate buffer (pH 7.4). The solubility of flavonoids was increased by the addition of methanol (10% *v*/*v*). The peak potentials in Table 1 and Figure 2 are indicated in mV (Ag/AgCl (saturated aqueous solution of KCl) reference electrode).

The differential pulse voltammogram of myricetin showed four peaks (Table 1, Figure 2). The very prominent peak 1 (−1 mV) corresponds to the oxidation of hydroxy groups of ring B [31]. The shape of this peak proposes that the electrochemical reaction can be followed by additional chemical reactions [32]. The hydroxy groups of ring C and ring A require a higher electrode potential. Especially, the *m*-dihydroxy group of ring A is probably oxidized at the potential of 798 mV (peak 5) due to their resorcinol arrangement which is less favorable for oxidation [33,34]. Additionally, the 3-hydroxy group and 5-hydroxy group are probably stabilized by a hydrogen bridge formation with the 4-keto group. Moreover, the redox potentials of both hydroxy groups are affected by a negative mesomeric effect of the 4-keto group as shown for the analogously structured quercetin, which only differs from myricetin by catechol arrangement of ring B [15,34,35].

The tricetin voltammogram showed five peaks (Table 1, Figure 2). Peak 1 appears at a potential of 72 mV (oxidation of hydroxy groups of ring B). Compared to myricetin, the oxidation requires a higher electrode potential. Furthermore, peak 1 shows an apparent shoulder at a potential of 175 mV (peak 2), which is only slightly visible in myricetin’s voltammogram. In the case of tricetin, the oxidation of the *m*-dihydroxy group of ring A required a higher potential (876 mV) than the oxidation of the *m*-dihydroxy group of myricetin ring A (798 mV).

### 2.2. Deoxyribose Degradation Assay

The deoxyribose degradation assay was performed in four variants. Each assay variant provides different information about the ROS scavenging abilities of tested substances [29]. 

#### 2.2.1. H_2_O_2_/Fe^III^EDTA/Ascorbic Acid Variant

**Figure 3:**
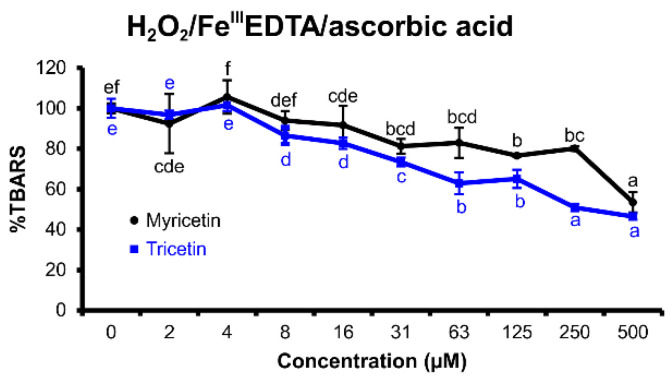
Thiobarbituric acid reactive species (TBARS) production in the reaction mixtures of the H_2_O_2_/Fe^III^EDTA/ascorbic acid deoxyribose degradation assay variant (1 h incubation time at 27 °C, pH 7.4) of myricetin (black) and tricetin (blue). (100% = TBARS of the control reaction mixture of the assay variant H_2_O_2_/Fe^III^EDTA/ascorbic acid). Error bars show standard deviation (SD) of three replicates; letters (a–f) indicate levels of significance (95% Duncan); EDTA = ethylenediaminetetraacetic acid.

This variant of the deoxyribose degradation assay (Figure 3) explores the ROS scavenging capability of the tested flavonoid, especially in terms of hydroxyl radicals or hydrogen peroxide, and its ability to inhibit the iron-catalyzed Fenton-like reaction. ROS scavenging decreases TBARS production that is caused by oxidative degradation of 2-deoxyribose [30]. The hydrogen peroxide presence simulates a scenario of increased oxidative stress with higher ROS concentrations due to mitochondria or tissue damage. Ascorbic acid, a strong reducing agent, acts as an iron redox cycling promotor [29,30]. Ethylenediaminetetraacetic acid (EDTA) is a potent transition metal chelator, which inhibits the formation of iron coordination complexes with the tested flavonoid in the reaction mixture of deoxyribose degradation assay. Therefore, the deoxyribose assay variant with Fe^III^EDTA allows exploring of the redox properties of the tested flavonoid independent of the electrochemical redox potential shifts that could be caused by coordination complex formation of iron(III) ions with the tested flavonoid [29,30].

Both tested flavonoids showed notable antioxidant effects, with tricetin being more efficient than myricetin. Myricetin was active between 31 and 500 μM, tricetin inhibited the oxidation of 2-deoxyribose up to a concentration of 8 μM.

#### 2.2.2. H_2_O_2_/Fe^III^EDTA Variant

If ascorbic acid is not added to the reaction mixture, the Fenton-like reaction has to be started by another reduction agent, in this case, the tested flavonoid, if it can reduce iron(III) to iron(II). Consequently, the pro-oxidant abilities of the tested flavonoid can be explored [29,30].

**Figure 4:**
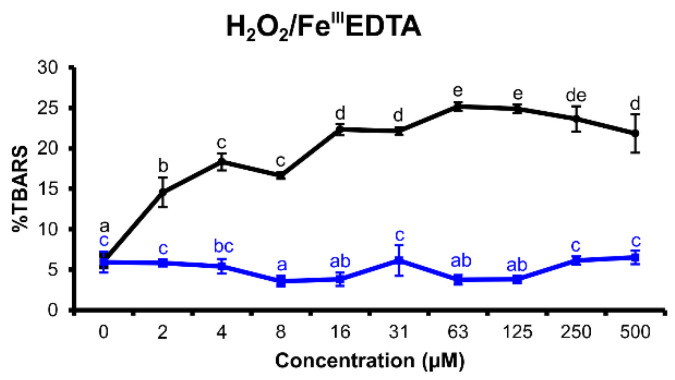
Thiobarbituric acid reactive species (TBARS) production in the reaction mixtures of the H_2_O_2_/Fe^III^EDTA deoxyribose degradation assay variant (1 h incubation time at 27 °C, pH 7.4) of myricetin (black) and tricetin (blue). (100% = TBARS of the control reaction mixture of the assay variant H_2_O_2_/Fe^III^EDTA/ascorbic acid). Error bars show standard deviation (SD) of three replicates; letters (a–e) indicate levels of significance (95% Duncan); EDTA = ethylenediaminetetraacetic acid.

In this assay variant (Figure 4), myricetin demonstrated weak pro-oxidant effects, which became evident between 2 and 500 μM. By contrast, tricetin showed no effects in the investigated concentration range.

#### 2.2.3. Fe^III^EDTA/Ascorbic Acid Variant

Ascorbic acid is a well-known reducing agent, which promotes iron redox cycling. Iron(II) can reduce molecular oxygen dissolved in the reaction mixture to superoxide anion radical, which relatively quickly dismutates to hydrogen peroxide. Therefore, the tested flavonoids can show either antioxidant effects if they scavenge ROS or pro-oxidant effects if they increase the redox cycling rate more effectively than ascorbic acid [29].

**Figure 5:**
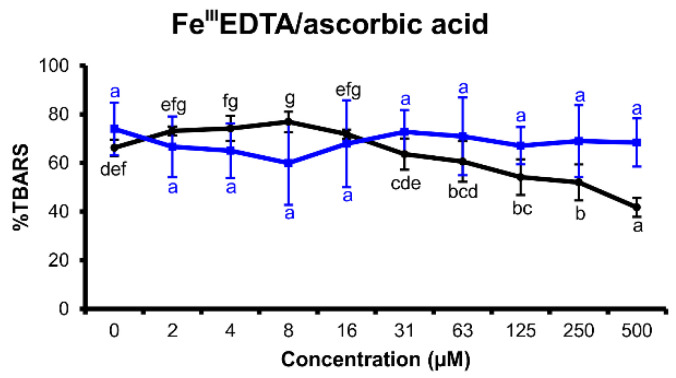
Thiobarbituric acid reactive species (TBARS) production in the reaction mixtures of the Fe^III^EDTA/ascorbic acid deoxyribose degradation assay variant (16 h incubation time at 27 °C, pH 7.4) of myricetin (black) and tricetin (blue). (100% = TBARS of the control reaction mixture of the assay variant H_2_O_2_/Fe^III^EDTA/ascorbic acid). Error bars show standard deviation (SD) of three replicates; letters (a–g) indicate levels of significance (95% Duncan); EDTA = ethylenediaminetetraacetic acid.

Myricetin inhibited the pro-oxidant effects of ascorbic acid (Figure 5). Nevertheless, this activity was very weak and only evident in the concentration range of 125 to 500 μM. Tricetin did not affect the oxidative degradation of 2-deoxyribose in this assay arrangement at all.

#### 2.2.4. Fe^III^EDTA Variant

**Figure 6:**
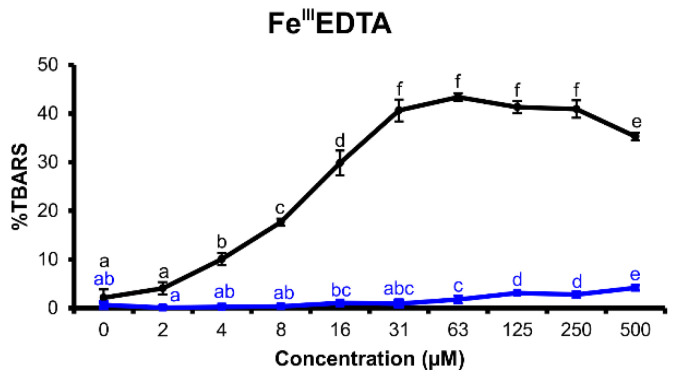
Thiobarbituric acid reactive species (TBARS) production in the reaction mixtures of the Fe^III^EDTA deoxyribose degradation assay variant (16 h incubation time at 27 °C, pH 7.4) of myricetin (black) and tricetin (blue). (100% = TBARS of the control reaction mixture of the assay variant H_2_O_2_/Fe^III^EDTA/ascorbic acid). Error bars show standard deviation (SD) of three replicates; letters (a–f) indicate levels of significance (95% Duncan); EDTA = ethylenediaminetetraacetic acid.

This variant of the assay explores if the investigated flavonoid is able to reduce iron(III) to iron(II) and promote iron redox cycling. Therefore, this assay variant investigates possible pro-oxidant activities of the tested flavonoids [29]. Both explored flavonoids appeared to support ROS production by iron redox cycling promotion (Figure 6), though myricetin showed a much more pronounced effect than tricetin.

## 3. Discussion

So-called antioxidants can also cause pro-oxidant effects by reducing molecular oxygen to ROS simultaneously to scavenging them, depending on the reaction equilibria. Additionally, the inhibition or promotion of iron redox cycling appears to constitute a further important factor, which can modulate pro- or antioxidant effects. The enhancement of iron redox cycling accelerates the Fenton-like reaction rate and increases cytotoxic hydroxyl radical concentrations [36].

The conclusions of differential pulse voltammetry correspond well with those from the deoxyribose degradation assay variants. The voltammogram indicates that myricetin and tricetin are strong reducing agents, particularly due to the relatively low electrode potentials required for their ring B hydroxy groups oxidation (Table 1; −1 mV and 72 mV respectively). Compared to the *o*-dihydroxy groups of flavonoid ring B, the pyrogallol moiety usually shows two signals on voltammograms [15,35,37]. The first peak indicates a formation of semiquinone, and the second one corresponds to the oxidation of the semiquinone (stabilized by the resonance of the flavonoid conjugated system [14]) to *o*-quinone [37]. Accordingly, peaks 1 and 3 (Figure 2) represent signals of gradual oxidation of the pyrogallol moiety. The peak potential of myricetin pyrogallol moiety oxidation was lower than that of tricetin (Figure 2). Comparted to tricetin’s voltammogram, myricetin’s voltammetric curve is missing an evident shoulder (peak 2); however, its peak 1 shows a tailing shoulder part probably due to a formation of other redox-active substances. Nevertheless, myricetin’s peak 1 shoulder and tricetin’s peak 2 signal probably indicate the existence of different processes for the two flavonoids on the electrode surface.

In comparison to tricetin, the cathodic shift of the myricetin trihydroxylated ring B oxidation was probably caused by a positive mesomeric effect of myricetin’s enolic 3-hydroxy group. The mesomeric effect increased electron density in the conjugated system, as demonstrated for the structurally similar flavonoids luteolin and quercetin [15]. Therefore, the oxidation became easier in the case of myricetin than in tricetin that lacks the enolic 3-hydroxy group. Albeit the hydroxy groups of ring B are the main target for redox reactions with ROS, the reactivity of other hydroxy groups, such as that on position 3 of ring C, should not be underestimated [14,33,35]. The peaks 5 of myricetin and tricetin are extremely broad and asymmetric, which indicates complex follow-up chemical reactions, probably a substitution [38] or even polymerization [39]. The enolic 3-hydroxy group clearly affects the electrode potential of resorcinol moiety oxidation due to conjugation of the rings A, B, and C [23,34]. Therefore, the redox potential required for oxidation of these hydroxy groups of tricetin is higher compared to that of myricetin. The differential pulse voltammetry proved that both tested flavonoids, myricetin and tricetin, are good reduction agents. However, because of the lower redox potential of the myricetin peaks, this flavonoid is a more potent reducing agent than tricetin, which is supported by the results of deoxyribose degradation assay variants.

In the H_2_O_2_/Fe^III^EDTA/ascorbic acid variant of deoxyribose degradation assay, myricetin and tricetin showed excellent antioxidant effects (Figure 3). Nevertheless, myricetin proved to be a more potent reducing agent than tricetin. Furthermore, myricetin was able to reduce iron(III) to iron(II) efficiently, which became evident both in the H_2_O_2_/Fe^III^EDTA and Fe^III^EDTA assay variants (Figure 4 and Figure 6). This observed characteristic of myricetin agrees with previous experimental results of Laughton et al. [40] and the voltammetric investigation of Jovanovic et al. [41]. Both antioxidant and pro-oxidant effects of myricetin were evident in the Fe^III^EDTA/ascorbic acid variant. The dose–response curve displays an inverted U-shape. This can be explained by an increased rate of iron redox cycling in the presence of ascorbic acid and myricetin. Knickle et al. proposed that myricetin’s pro-oxidant properties are responsible for selective apoptosis induction in the cells of triple-negative breast cancer cell lines [42]. However, in higher concentrations, myricetin may again scavenge the formed hydroxyl radicals. The interactions of tested flavonoids with ascorbic acid reactions inform about a possible inhibition of ascorbic acid’s pro-oxidant effects. Ascorbic acid, a reducing agent accumulated in some organs such as the brain (up to concentrations 1 mM) [43], can release iron ions from ferritin and increase tissue oxidative stress [44]. Since oxidative stress is associated with many degenerative diseases such as multiple sclerosis [45] or Parkinson’s disease [8], understanding the interaction harmony of various redox-active agents becomes important. Nevertheless, real cell protoplast contains a complicated mixture of various substances. Therefore, in vitro assay experiments can be useful to better understand in vivo assay results.

Albeit flavonoid bioavailability from the human gut and their ability to penetrate through the blood–brain barrier (BBB) is extremely limited, they still can positively affect functions of the vascular or immune system and the brain-gut axis [46]. For cellular redox equilibria maintenance, low concentrations of flavonoids are probably sufficient. In patients suffering from neurodegenerative diseases, the BBB is often damaged [47], and this may increase flavonoid penetration from blood to brain tissues. Therefore, the redox activity of flavonoids can beneficially affect ROS levels in various tissues.

## 4. Materials and Methods

### 4.1. Chemicals

Tricetin was obtained from Extrasynthese (Genay Cedex, France). Myricetin and all other chemicals and organic solvents were purchased from Sigma-Aldrich (St. Louis, MO, USA). All chemicals and organic solvents were of analytical grade. The water was of Milli-Q quality (Milli-Q Advantage A10 System, Milllipore SAS, Molsheim, France).

### 4.2. Differential Pulse Voltammetry

Voltammetric curves were recorded with a three-electrode system, µAutolab PGSTAT type III (EcoChemie Inc., Utrecht, The Netherlands). The working electrode was a glassy carbon electrode of 3 mm diameter (Metrohm AG, Herisau, Switzerland), an Ag/AgCl (saturated KCl) electrode was used as reference and platinum wire as a counter electrode. The glassy carbon electrode was cleaned with methanol and water and polished by aluminum oxide powder of 0.3 µm grain size (Metrohm AG, Herisau, Switzerland) before every measurement. The effective scan rate of the voltammetry was 21 mV/s, modulation time was 0.05 s, modulation amplitude was 25 mV, and the scan potential was from −250 to +1200 mV. One milliliter of 1 mM solution of the tested flavonoids in degassed methanol was mixed with 9 mL of the degassed buffer (0.1 M phosphate buffer pH 7.4). All electrolyte solutions were degassed by argon for 10 min. The measurements were performed under an argon atmosphere at room temperature (20 °C).

### 4.3. Deoxyribose Degradation Assay

The variants of the assay follow procedures, the detailed reaction mechanisms of which are described elsewhere [21]. The flavonoids were dissolved in different concentrations (0−500 µM) in an aqueous KH_2_PO_4_/KOH buffer solution (30 mM, pH 7.4); to 125 µL of this solution, 25 µL of a 10.4 mM 2-deoxyribose solution in the same buffer system and 50 µL of Fe^III^EDTA solution (50 µM) were added. The 50 µL contained 52 µM EDTA dissolved in buffer, which was premixed with the aqueous FeCl_3_ solution (1:1 *v*/*v*). Furthermore, 25 µL of 10.0 mM H_2_O_2_ in water and 25 µL of 1.0 mM ascorbic acid in the buffer were added in the case of H_2_O_2_/Fe^III^EDTA/ascorbic acid assay variant. In the other deoxyribose degradation assay systems, H_2_O_2_ or ascorbic acid was replaced by water or buffer, respectively. The temperature during incubation was 27 °C. The assay variants with H_2_O_2_ were incubated for 1 h; the systems without H_2_O_2_ were incubated for 16 h. After reaction with thiobarbituric acid and subsequent extraction of the pink pigment with 1-butanol, oxidative degradation products of 2-deoxyribose, thiobarbituric acid reactive species were determined photometrically at 532 nm (Tecan Infinite M200, Männedorf, Switzerland). The positive control (100% TBARS) was the H_2_O_2_/Fe^III^EDTA/ascorbic acid reaction mixture without the test compound. The blank contained the full reaction mixture except for 2-deoxyribose and was determined in each performed experiment series. Assays were performed in triplicate.

### 4.4. Statistics

One-way ANOVA with 95% Duncan’s multiple range test was used to determine relevant differences between various concentrations of the tested flavonoid in the deoxyribose degradation assay.

## 5. Conclusions

Flavonoids represent a group of natural plant constituents that can help to develop new perspective drugs against various diseases associated with aging or degeneration. However, minor changes in their structures can dramatically affect the antioxidant activity of flavonoids. The structurally similar flavonoids myricetin and tricetin serve as a good example. Compared to tricetin, the more efficient reducing properties of myricetin are probably caused by the positive mesomeric effect of enolic 3-hydroxy group on ring C. Tricetin, which is lacking this enolic group, caused mostly antioxidant effects or was inactive in the deoxyribose degradation assay.

## Figures and Tables

**Table 1:** Peak potentials of flavonoids myricetin and tricetin detected by differential pulse voltammetry. 0.1 mM flavonoid solution in 0.1 M phosphate buffer (pH 7.4) with 10% (*v*/*v*) methanol. (Glassy carbon working electrode; platinum counter electrode; Ag/AgCl (saturated aqueous KCl solution) reference electrode; effective scan rate: 21 mV/s; modulation time: 0.05 s; modulation amplitude: 25 mV).

Peak Number	Myricetin (mV)	Tricetin (mV)
1	−1	72
2	shoulder	175
3	191	316
4	513	575
5	798	876
